# Using Lactobacilli to Fight *Escherichia coli* and *Staphylococcus aureus* Biofilms on Urinary Tract Devices

**DOI:** 10.3390/antibiotics10121525

**Published:** 2021-12-14

**Authors:** Fábio M. Carvalho, Filipe J. M. Mergulhão, Luciana C. Gomes

**Affiliations:** LEPABE—Laboratory for Process Engineering, Environment, Biotechnology and Energy, Faculty of Engineering, University of Porto, Rua Dr. Roberto Frias, 4200-465 Porto, Portugal; up201502963@fe.up.pt (F.M.C.); filipem@fe.up.pt (F.J.M.M.)

**Keywords:** biofilm, urinary tract devices, probiotics, *Lactobacillus plantarum*, *Lactobacillus rhamnosus*, antibiofilm activity, displacement

## Abstract

The low efficacy of conventional treatments and the interest in finding natural-based approaches to counteract biofilm development on urinary tract devices have promoted the research on probiotics. This work evaluated the ability of two probiotic strains, *Lactobacillus plantarum* and *Lactobacillus rhamnosus*, in displacing pre-formed biofilms of *Escherichia coli* and *Staphylococcus aureus* from medical-grade silicone. Single-species biofilms of 24 h were placed in contact with each probiotic suspension for 6 h and 24 h, and the reductions in biofilm cell culturability and total biomass were monitored by counting colony-forming units and crystal violet assay, respectively. Both probiotics significantly reduced the culturability of *E. coli* and *S. aureus* biofilms, mainly after 24 h of exposure, with reduction percentages of 70% and 77% for *L. plantarum* and 76% and 63% for *L. rhamnosus*, respectively. Additionally, the amount of *E. coli* biofilm determined by CV staining was maintained approximately constant after 6 h of probiotic contact and significantly reduced up to 67% after 24 h. For *S. aureus*, only *L. rhamnosus* caused a significant effect on biofilm amount after 6 h of treatment. Hence, this study demonstrated the potential of lactobacilli to control the development of pre-established uropathogenic biofilms.

## 1. Introduction

Urinary tract infections (UTIs) are the most common type of healthcare-associated infections reported by the Centers for Disease Control [1,2,3], with an estimated annual worldwide incidence of 250 million cases [4]. The high incidence of these infections results in considerable treatment costs, increased length of hospital stays, and high mortality rates, posing a huge financial burden on healthcare systems [5,6,7,8]. Device-associated UTIs, caused by the insertion of urological devices (UDs), such as urinary catheters or ureteral stents, contribute to about 97% of UTIs [3,5,9]. Despite the efforts to maintain sterility, the contamination of UDs is almost inevitable since they work as a bridge connecting the nonsterile external environment and the patient’s body [10,11]. The most common microorganisms contributing to device-associated UTIs are *Escherichia coli*, *Klebsiella pneumoniae*, *Pseudomonas aeruginosa*, *Staphylococcus aureus*, *Candida* spp., *Enterococcus faecalis*, and *Proteus mirabilis* [12,13,14,15].

Device-associated UTIs mostly originate from the formation of microbial pathogenic biofilms on the device’s surface [16]. Biofilms are present in about 80% of human microbial infections [12], and once established, they are extremely hard to eliminate [17,18]. Biofilms are defined as a consortium of microorganisms surrounded by a self-synthesized matrix of extracellular polymeric substances [19,20], which protects the embedded bacteria against host defenses and antimicrobial agents [12]. The conditions present in the urinary tract are particularly favorable to microbial adhesion and biofilm development due to the diversity of shear forces prevailing throughout the urinary tract [5,21], the presence of a continuous or intermittent flow of nutrients [12], the absence of defense mechanisms at the UD lumen [12], and the high vulnerability of UDs, classically made of polymeric materials, to bacterial adhesion [22]. In addition, bacteria such as *P. mirabilis* can cause the precipitation of some minerals present in urine, which originates encrustation on UD surfaces and has severe consequences on the bladder and urethral epithelia [9,12].

Some strategies to control UD biofilms include antimicrobial lubricants, bladder instillation or irrigation, antimicrobial agents in collection bags, and the administration of antibiotics [13]. Recently, in a novel approach, the balloon on Foley catheters was transformed into a permeable membrane allowing localized and continuous delivery of antibiotics to the bladder and was shown to eradicate a provoked uropathogenic *E. coli* infection [23]. Moreover, the development of new surfaces that inhibit biofilm formation through antimicrobial agent’s release, contact-killing, inhibition of microbial adhesion, and the disruption of biofilm architecture have been suggested to reduce the incidence of device-associated UTIs [24,25]. Although numerous strategies have been investigated, questions regarding biocompatibility, bacterial resistance, long-term efficacy, and cytotoxicity warrants further investigation, not being clear how they will affect clinical outcomes [26].

Recent evidence suggests probiotics as a promising option for fighting biofilms. Probiotics are defined as “live microorganisms which when administered in adequate amounts confer a health benefit on the host” [27]. Lactic acid bacteria (LAB), including *Lactobacillus*, *Bifidobacterium*, *Streptococcus*, *Lactococcus*, and *Leuconostoc*, are the predominant group of bacteria with proven probiotic action [28,29], where *Lactobacillus* assume the greatest relevance [30]. This group of bacteria can grow in different habitats using diverse sources of carbon [31]. From glucose metabolism, LAB are classified as homofermentative, producing exclusively lactic acid, or heterofermentative, producing several other metabolites besides lactic acid, such as ethanol and acetic acid [32,33]. Those substances, together with other secondary metabolites, such as organic acids, exopolysaccharides, biosurfactants, bacteriocins, and enzymes [34], provide a physiologically restrictive environment (e.g., low pH, redox potential, hydrogen sulfide, and peroxide production), making it less suitable for competitors [35,36,37]. Bacteriocins are a particular class of exometabolites produced by probiotics and are substantially documented to inhibit the growth of competitors [38,39,40]. Probiotics can also compete for adhesion sites by forming non-pathogenic biofilms that hamper the adhesion and biofilm formation of pathogens [41,42]. Each probiotic strain has multiple and diverse impacts on the host [36]. To date, several studies demonstrated the ability of probiotics to produce antimicrobial metabolites to manage biofilm infections [43,44,45], and their inhibitory effects on biofilm formation were extensively reviewed [46,47,48]. Moreover, probiotics were described to suppress quorum-sensing and affect biofilm integrity [46] by repressing the expression of biofilm-associated genes [49].

The objective of this study was to evaluate the effect of two *Lactobacillus* strains frequently used in antimicrobial studies, *Lactobacillus plantarum* (currently *Lactiplantibacillus plantarum*) and *Lactobacillus rhamnosus* (currently *Lacticaseibacillus rhamnosus*), against pre-formed biofilms of bacteria commonly found in biofilms developed in UDs, *E. coli* and *S. aureus*. Some studies showed promising results in displacing adhering uropathogens from catheter materials [16,50,51]. However, to the best of our knowledge, this is the first study that demonstrates the ability of probiotic cells to displace pre-formed biofilms combining the effect of nutritional conditions, temperature, hydrodynamics, and surface material to better predict how probiotics will perform in vivo.

## 2. Results

To evaluate the capacity of probiotics to disrupt pre-formed pathogenic biofilms, a dynamic biofilm assay was performed where the cell culturability and total biomass were analyzed by colony-forming unit (CFU) count and crystal violet (CV) staining, respectively.

### 2.1. Biofilm Cell Culturability

**Figure 1:**
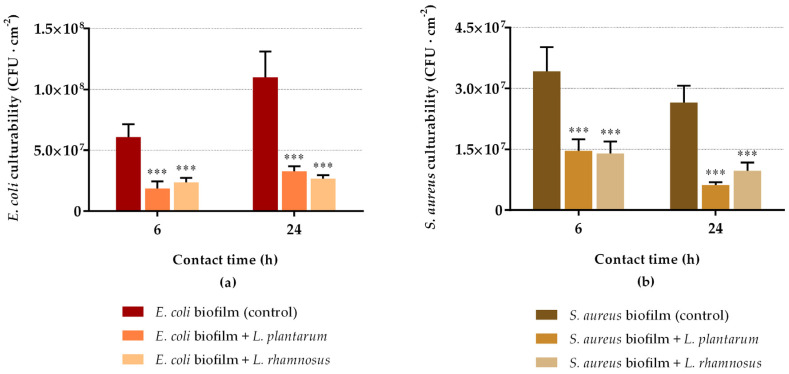
Culturability of 24 h biofilms of *E. coli* (**a**) and *S. aureus* (**b**) after 6 h and 24 h of contact with probiotics (*L. plantarum* or *L. rhamnosus*). The data present the mean and standard deviation (SD) of at least three independent experiments. Statistically significant differences between the control and treatment were considered for *p*-values < 0.05 (*** indicates *p* < 0.001).

The results of *E. coli* and *S. aureus* biofilm culturable cells after 6 h and 24 h of incubation with *L. plantarum* and *L. rhamnosus* suspensions are presented in Figure 1. Both model pathogenic strains confirmed their ability to grow in artificial urine medium, as well as to adhere and form stable biofilms on silicone rubber. The ability of *E. coli* to form robust biofilms on this surface material was previously reported by our research group [52,53]. Moreover, *E. coli* biofilms exhibited higher cellular densities than *S. aureus* (Figure 1), demonstrating its higher propensity to form biofilms on silicone.

Both *Lactobacillus* strains were able to reduce pre-formed biofilms. As regards *E. coli* biofilms (Figure 1a), the number of sessile culturable cells was significantly reduced when exposed to probiotics in comparison to the negative control sample (*p* < 0.001). The highest reductions were obtained after 24 h of exposure, with reductions of 70% for *L. plantarum* and 76% for *L. rhamnosus* (*p* < 0.001). Additionally, reductions of 69% and 61% were obtained after 6 h of biofilm treatment with *L. plantarum* and *L. rhamnosus*, respectively (*p* < 0.001). Regarding *S. aureus* biofilms (Figure 1b), the same tendency was observed. There was a significant reduction in *S. aureus* culturability at both experimental times (*p* < 0.001), being the most significant decrease after 24 h of contact (77% for *L. plantarum* and 63% for *L. rhamnosus*); after 6 h, reductions of 57% and 59% were obtained for *L. plantarum* and *L. rhamnosus*, respectively. For both pathogens, the antimicrobial activity of probiotics increased from 6 h to 24 h of exposure, demonstrating a progressive effect over time.

**Figure 2:**
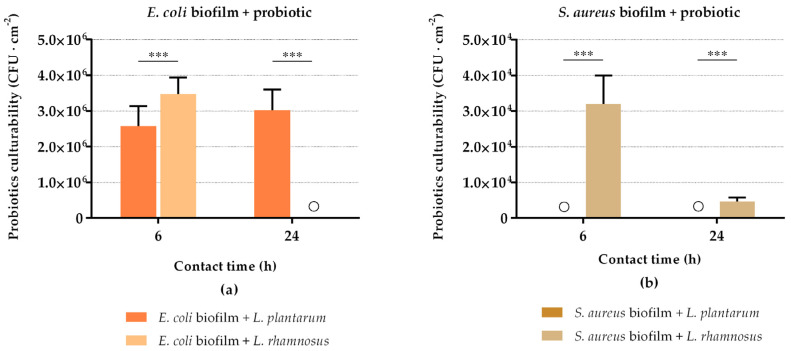
Culturability of attached *L. plantarum* and *L. rhamnosus* cells after 6 h and 24 h of contact with *E. coli* (**a**) and *S. aureus* (**b**) biofilms. The data present the mean and SD of at least three independent experiments. Statistically significant differences between probiotic strains for each time point were considered for *p*-values < 0.05 (*** indicates *p* < 0.001). “◯” indicates that no colonies were detected.

The culturability of probiotics in the sessile state was simultaneously evaluated (Figure 2). It can be observed that the *E. coli* culturability reduction (Figure 1a) was accompanied by the presence of viable probiotic cells in the biofilms (Figure 2a), except for *L. rhamnosus*, which lost its biofilm culturability after 24 h of interaction with *E. coli*. On the contrary, the reduction in *S. aureus* biofilm culturability (Figure 1b) was not followed by the presence of viable *L. plantarum* cells in biofilms (no colonies were detected at 6 h and 24 h; Figure 2a). *L. rhamnosus* was present in sessile conditions, but its culturability decreased by 85% between 6 h and 24 h of interaction with *S. aureus* (Figure 2b). Additionally, looking at Figure 1 and Figure 2, there is about 1–3 log CFU·cm^−2^ difference between the populations of *E. coli* and *S. aureus* and probiotics within the biofilms.

Complementary assays were performed to analyze the presence of probiotic cells in the planktonic fraction in order to explain the decrease in pathogens culturability, even in the absence of *Lactobacillus* cells in the biofilm (data not shown). For example, *L. rhamnosus* cell densities of 2.5 × 10^5^ ± 4.3 × 10^3^ and 1.6 × 10^4^ ± 4.2 × 10^3^ CFU·mL^−1^ were detected in the suspension after 24 h of contact with *E. coli* and *S. aureus* biofilms, respectively, suggesting that probiotics may act on the pathogenic sessile cells through the release of harmful substances to the extracellular medium. The antimicrobial activity of lactobacilli was also evaluated against *S. aureus* by the disk diffusion method (Supplementary Material). The cell suspensions and cell-free supernatants of both viable and lysed probiotics evidenced clear inhibition zones on the swabbed *S. aureus* on Luria-Bertani agar plates when compared with the negative control (Table S1, Supplementary Material), indicating that these probiotic strains inhibited the growth of pathogens through the production and secretion of antimicrobial substances into their surroundings.

### 2.2. Biofilm Mass

**Figure 3:**
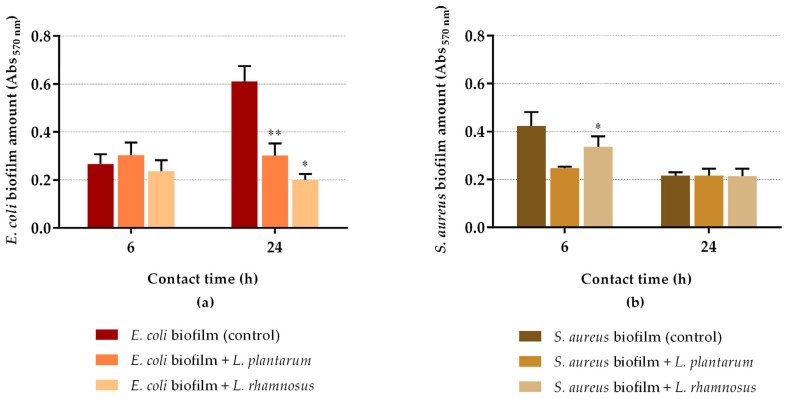
Total amount of *E. coli* (**a**) and *S. aureus* (**b**) biofilms after 6 h and 24 h of contact with probiotics (*L. plantarum* or *L. rhamnosus*). The data present the mean and SD of at least three independent experiments. Statistically significant differences between the control and treatment were considered for *p*-values < 0.05 (* and ** indicate *p* < 0.05 and *p* < 0.01, respectively).

Figure 3 presents the results of biofilm quantification using CV staining. Concerning *E. coli* biofilms (Figure 3a), both probiotics maintained the biofilm amount at 6 h of contact in comparison with control (*p* > 0.05). However, after 24 h of exposure, *L. plantarum* and *L. rhamnosus* significantly reduced the biofilm mass by 51% (*p* = 0.04) and 67% (*p* = 0.011), respectively, demonstrating their capacity to disrupt the pre-formed biofilms. In the case of *S. aureus* biofilms, the opposite behavior was observed. After 6 h of contact, both probiotics reduced biofilm mass, although only *L. rhamnosus* exhibited statistical difference when compared to control (reduction of 42% for *L. plantarum* (*p* = 0.053) and 20% for *L. rhamnosus* (*p* = 0.049)); at 24 h of contact, the biofilm amount remained nearly constant. Therefore, there was a poor correlation between the CV staining method and cell culturability.

## 3. Discussion

Device-associated urinary tract infections are a critical problem caused by the high propensity of medical devices to microbial colonization. Previous studies have proposed the use of probiotics as a useful strategy to control pathogenic biofilms, demonstrating that probiotics cells and metabolites can displace adhering uropathogens from urinary devices materials and block bacterial adhesion to uroepithelial cells [47,48]. Probiotics can exert their antibiofilm activity by adopting different strategies: displacement, exclusion, and competition [47]. Recently, our research group evaluated the ability of *L. plantarum* biofilms to prevent *E. coli* adhesion and biofilm formation on silicone rubber, following an exclusion strategy [42]. In the present work, the potential of two probiotic strains (*L. plantarum* and *L. rhamnosus*) to disperse pre-formed biofilms of *E. coli* and *S. aureus* under physiologically relevant conditions was assessed by adopting a displacement strategy [48].

Regarding the antibiofilm activity of probiotics, they significantly inhibited the proliferation of *E. coli* and *S. aureus* biofilms by reducing their cell culturability and biomass amount after 24 h of treatment. Furthermore, both *Lactobacillus* strains caused similar reductions in the culturability of both model pathogens. The activity of probiotics can be related to interfacial cell–cell interactions and to the production and release of antagonizing metabolites that are able to destabilize the biofilm organization, as demonstrated in previous studies [54,55,56]. Although the incorporation of probiotics within the biofilms suggests that those adhered *Lactobacillus* cells may contribute to pathogen inhibition, the absence of biofilm culturable cells of probiotics at some time points (*L. rhamnosus* after 24 h of contact with *E. coli* and *L. plantarum* after 6 h and 24 h of contact with *S. aureus*) suggests that, beyond the antibiofilm action by integration and contact with the biofilm, lactobacilli may act through the release of antimicrobial substances from the planktonic cells. Moreover, the differences found between the culturability of *E. coli* and *S. aureus* and the culturability of probiotics mean that there was not a direct exchange of pathogenic cells by probiotic cells during treatment. This suggests that pathogen inactivation occurred essentially by the secretion of antimicrobial substances into the surrounding environment. The presence of culturable cells of probiotics in the planktonic fraction and the existence of inhibition zones caused by probiotics cell-free supernatants reinforce this hypothesis.

The poor correlation between the crystal violet (CV) method and cell culturability results can be attributed to the ability of CV to non-specifically bind to some components of the biofilm matrix (such as DNA, exopolysaccharides (EPS), and proteinaceous material) and the peptidoglycan wall of both live and dead cells [57,58].

The antimicrobial substances released by probiotics may penetrate the extracellular matrix of biofilms, interfering with their integrity and cell culturability. Several authors reported the ability of *Lactobacillus* strains to disrupt mature biofilms. Jaffar et al. [58] described that *L. plantarum* significantly disrupted the pre-formed biofilm of *Aggregatibacter actinomycetemcomitans* by approximately 61% on polystyrene after 24 h. Conversely, Song et al. [59] reported that *L. rhamnosus* was capable of disrupting biofilms of *Candida albicans* by 99.9% due to the production of lactic acid and antimicrobial peptides, which presumably disrupted the cytoplasmatic membrane and inactivated cytoplasm molecules. This mechanism was supported by Fayol-Messaoudi et al. [60], who suggested that the bactericidal activity of *Lactobacillus* strains may be due to the synergistic action of lactic acid and secreted bacteriocins. Some studies have shown that bacteriocins produced by *L. plantarum* effectively suppressed the growth and biofilm formation of several microorganisms [39,61,62], including *S. aureus* [63]. McMillan et al. [64] found that *L. rhamnosus* was incorporated into uropathogenic biofilms, including *E. coli*, and caused significant *E. coli* killing supposedly due to the production of bacteriocins or biosurfactant-like substances. In agreement, Cadieux et al. [65] described that *L. rhamnosus* strongly inhibited the growth of uropathogenic *E. coli* by producing bacteriocins, hydrogen peroxide, and lactic acid. Similarly, Otero et al. [45] evidenced an inhibitory effect of *Lactobacillus* strains in *S. aureus* growth after 6 h of co-culture, possibly due to the combined effect of hydrogen peroxide and lactic acid. In addition, some authors suggested that *L. rhamnosus* can produce biosurfactants with antibiofilm activity against *E. coli* and *S. aureus* [43,64]. Previous studies have demonstrated the ability of lactic acid bacteria biosurfactants to inhibit biofilm development and induce its dispersion from surgical implant materials [66,67,68]. Furthermore, an assorted number of studies attributed the disruption of the architecture of pathogenic biofilms by *L. rhamnosus* to the secretion of molecules that downregulate the genes involved in biofilm development, DNA replication, translation, glycolysis, and gluconeogenesis [55,56]. Song et al. [69] reported that *L. rhamnosus* significantly disrupted the architecture of *E. coli* biofilms by 82% by decreasing the transcriptional activity of transcriptional activators (*luxS*, *lsrK*, and *lsrR*) of the quorum-sensing in *E. coli*. In addition, Ahn et al. [70] and Kim et al. [71] described that lipoteichoic acid produced by *L. plantarum* disrupted pre-formed biofilms of single and multispecies pathogens by interfering with EPS production.

In this work, the lack of glucose in the growth medium hindered probiotics fermentation since high glucose concentrations enhance medium acidification [72,73]. This was confirmed by comparing the initial and final pH values of artificial urine medium (AUM), where no differences were found. Additionally, Todorov et al. [74] studied the effect of the pH on the production of bacteriocins of *L. plantarum* and found that the inhibitory activity of bacteriocins was detected at pH values between 5.0 and 6.5. Moreover, the presence of yeast extract, potassium dihydrogen phosphate, and dipotassium hydrogen phosphate in culture medium as nitrogen and phosphorus sources, respectively, enhanced the production of bacteriocins by *L. plantarum* [74]. In the present work, since the initial pH of AUM is 6.5, and yeast extract, potassium dihydrogen phosphate, and dipotassium hydrogen phosphate are present in AUM [75], this may have contributed to the production of bacteriocins by this probiotic strain. Regarding biosurfactants, despite the bactericidal potential of biosurfactants, their action is more frequently associated with their capacity to affect microbial adhesion by interfering with surface tension and hydrophobicity [76,77], so biosurfactants are unlikely to be effective on mature biofilms. Thus, the disruptive activity of probiotics is most likely explained by the production and secretion of antimicrobial exometabolites, such as hydrogen peroxide, bacteriocins, and biosurfactants, rather than by a possible pH change of the culture media.

Overall, these results suggest that the ability of probiotics to displace pre-formed uropathogenic biofilms can be attributed to the production of exometabolites that inactivate sessile cells and destabilize the biofilm structure.

## 4. Materials and Methods

### 4.1. Preparation of Silicone Surfaces

Biofilm formation experiments were performed on silicone coupons (1 × 1 cm; Neves & Neves, Lda, Maia, Portugal) with the intention of mimicking the most common material of urinary catheters [78]. The surfaces were prepared as previously described by Carvalho et al. [42]. Briefly, the coupons were washed with 70% (*v*/*v*) ethanol (VWR, Radnor, PA, USA), air-dried, and then sterilized through ultra-violet (UV) radiation for 30 min. The sterile coupons were fixed to the bottom of 12-well polystyrene plates (VWR, USA) using double-sided adhesive tape, which was sterilized beforehand for 30 min through UV radiation.

### 4.2. Bacterial Strains and Culture Conditions

The probiotic strains used in this study were *Lactobacillus plantarum* and *Lactobacillus rhamnosus* (1 × 10^11^ CFU·g^−1^; Biomodics ApS, Rødovre, Denmark). These bacteria were preserved at −80 °C in De Man, Rogosa and Sharpe (MRS) broth (Merck KGaA, Madrid, Spain) with 30% (*v*/*v*) glycerol, streaked on MRS agar (Scharlab, S.L., Barcelona, Spain) plates, and incubated for 48 h at 37 °C. *Lactobacillus* inocula were prepared by collecting bacterial colonies from the MRS agar plate into 250 mL of MRS broth and incubating overnight at 37 °C in an orbital shaker at 120 rpm (Agitorb 200, Aralab, Rio de Mouro, Portugal). MRS is the culture media routinely used for lactobacilli growth [79].

*Escherichia coli* CECT 434 and *Staphylococcus aureus* CECT 976 were chosen as model microorganisms of biofilm-based urinary tract infections. These bacterial strains were preserved at −80 °C in Luria-Bertani (LB) broth (Thermo Fisher Scientific, Waltham, MA, USA) containing 30% (*v*/*v*) glycerol, streaked on LB agar plates, and incubated for 24 h at 37 °C. The starting cultures were prepared by collecting single colonies from LB agar plates to 250 mL of artificial urine medium (AUM) [75] and incubating overnight at 37 °C and 120 rpm. AUM was used to simulate the nutrient composition of human urine [75].

### 4.3. Influence of Probiotics on Pre-Formed Biofilms

The antibiofilm assays followed a displacement strategy [47], which consisted of the formation of *E. coli* and *S. aureus* biofilms, after which the biofilms were inoculated with the *Lactobacillus* strains separately in order to evaluate their ability to disperse pre-formed biofilms. The bacterial cultures grown overnight were harvested by centrifugation at 3202× *g* for 10 min at 25 °C (Eppendorf Centrifuge 5810R, Hamburg, Germany), and the final cell concentration was adjusted in fresh AUM to an optical density at 610 nm of 0.15 for *E. coli*, 0.20 for *S. aureus*, and 0.70 for both *Lactobacillus* strains, equivalent to approximately 10^8^ CFU·mL^−1^, the recommended bacterial density to be used in urological experiments [5]. This was confirmed by colony-forming unit (CFU) counts. The uropathogenic biofilms were formed on silicone coupons placed inside 12-well plates where each well was filled with 3 mL of the respective pathogenic suspension. The plates were incubated for 24 h at 37 °C under shaking conditions in order to generate shear stresses similar to those found inside urinary catheters [42,80]. Afterwards, cell suspensions were removed, and each well was loaded with 3 mL of the respective probiotic suspension for periods of contact of 6 h and 24 h under the same growth conditions. A negative control was prepared by adding sterile AUM to pathogenic biofilms. At the end of each experimental period, biofilms were analyzed as previously described [42]. Briefly, cell suspensions were removed from the wells, the non-adherent cells were washed with a sodium chloride solution (8.5 g·L^−1^ NaCl), and the biofilm amount and culturability were determined by crystal violet (CV) staining and CFU counts, respectively. All experiments included at least three independent biological replicates with two technical replicates each.

#### 4.3.1. Bacterial Enumeration

The number of biofilm culturable cells per cm^2^ of silicone was determined as indicated by Carvalho et al. [42]. Briefly, the coupons were transferred to Falcon tubes with 2 mL of saline solution and the biofilm cells were detached from the coupons by vortexing (ZX4, Velp Scientifica, Usmate Velate, Italy) for 2 min at full power. Then, serial dilutions of the obtained biofilm cell suspensions were performed in saline solution, plated on LB agar (selective media for *E. coli* and *S. aureus*) and MRS agar (selective media for *L. plantarum* and *L. rhamnosus*), and incubated at 37 °C for 24 h and 48 h, respectively.

The percentages of CFU reduction in pathogens were estimated as follows:

(1) $$\mathrm{Reduction}\;\left(\%\right)=\frac{{\mathrm{CFU}}_{\mathrm{control}}-{\mathrm{CFU}}_{\mathrm{biofilm}}}{{\mathrm{CFU}}_{\mathrm{control}}}\times 100$$

where CFU_control_ corresponds to the number of culturable cells of pathogens in the negative control samples (biofilms not exposed to probiotic cell suspensions), and CFU_biofilm_ is the number of culturable cells in biofilms treated with probiotics.

#### 4.3.2. Biofilm Amount Determination

The total mass of biofilms was quantified using the CV staining method [42]. Briefly, after washing the non-adherent cells, silicone coupons were transferred to 24-well polystyrene plates (Thermo Fisher Scientific, USA), and biofilms were fixed with 1 mL of 100% ethanol (VWR, USA) for 15 min. Then, the wells were air-dried, and biofilms were stained with 1 mL of 1% (*v*/*v*) CV (Merck, Germany) solution for 5 min. The dye bounded to the biofilm was solubilized by adding 1 mL of 33% (*v*/*v*) acetic acid (VWR, USA) solution. Finally, 200 μL of each well was transferred to a 96-well polystyrene plate (VWR, USA), and the biofilm mass was determined through absorbance measurement at 570 nm (Abs_570 nm_) in a microtiter plate reader (SpectroStar Nano, Biogen Cientifica S. L., Madrid, Spain). When the absorbance values exceeded 1, samples were diluted in 33% (*v*/*v*) acetic acid. The biofilm amount was expressed as Abs_570 nm_ values.

### 4.4. Statistical Analysis

Statistical analysis was performed using the IBM SPSS Statistics version 26 for Windows (IBM SPSS, Inc., Chicago, IL, USA). Descriptive statistics were used to calculate the mean and standard deviation (SD) for the number of culturable cells and biofilm mass. The homogeneity of variances and normality of data were verified for all response variables tested using the Kolmogorov–Smirnov and Shapiro–Wilk tests. Since the response variables were not normally distributed, a nonparametric analysis using the Kruskal–Wallis test was performed to assess whether there were statistically significant differences among groups, and the differences between those groups were determined by the Mann–Whitney test. Statistically significant differences were considered for *p*-values < 0.05, corresponding to a confidence level of 95% (*, **, and *** indicate *p* < 0.05, *p* < 0.01 and *p* < 0.001, respectively). All reported data are presented as mean ± SD from at least three experiments with duplicates.

## 5. Conclusions

*L. plantarum* and *L. rhamnosus* were able to displace pre-established biofilms of *E. coli* and *S. aureus* at similar levels. The antibiofilm activity of these probiotic strains may be primarily linked to the release of self-produced substances, which reduced the number of culturable pathogens in biofilms. Additionally, the integration of probiotic cells into the biofilm may have contributed to the destabilization of the biofilm organization.

This proof-of-principle study supports the potential of *Lactobacillus* strains to be used as biocontrol agents against pathogenic biofilms developed on urinary tract devices. It will pave the way for more experiments on the topic in an effort to elucidate the mechanisms underlying the lactobacilli activity.

## Data Availability

The data presented in this study are available on request from the corresponding author. The data are not publicly available yet as some data sets are being used for additional publications.

## Supplementary Materials

The following are available online at https://www.mdpi.com/article/10.3390/antibiotics10121525/s1. Detection of Antimicrobial Compounds by Growth Inhibition on Agar Plate; Table S1: The inhibitory activity of viable and lysed *L. plantarum* and *L. rhamnosus* suspensions and cell-free supernatants on the growth of *S. aureus*.

## References

[1] Centers for Disease Control and Prevention Catheter-associated Urinary Tract Infections (CAUTI)|HAI|CDC. https://www.cdc.gov/hai/ca_uti/uti.html.

[2] Siddiq D.M., Darouiche R.O. (2012). New strategies to prevent catheter-associated urinary tract infections. Nat. Rev. Urol..

[3] Maharjan G., Khadka P., Siddhi Shilpakar G., Chapagain G., Dhungana G.R. (2018). Catheter-Associated Urinary Tract Infection and Obstinate Biofilm Producers. Can. J. Infect. Dis. Med. Microbiol..

[4] (2011). World Health Organization Report on the Burden of Endemic Health Care-Associated Infection Worldwide. https://apps.who.int/iris/handle/10665/80135.

[5] Ramstedt M., Ribeiro I.A.C., Bujdakova H., Mergulhão F.J.M., Jordao L., Thomsen P., Alm M., Burmølle M., Vladkova T., Can F. (2019). Evaluating efficacy of antimicrobial and antifouling materials for urinary tract medical devices: Challenges and recommendations. Macromol. Biosci..

[6] Percival S.L., Suleman L., Vuotto C., Donelli G. (2015). Healthcare-associated infections, medical devices and biofilms: Risk, tolerance and control. J. Med. Microbiol..

[7] Khatoon Z., McTiernan C.D., Suuronen E.J., Mah T.F., Alarcon E.I. (2018). Bacterial biofilm formation on implantable devices and approaches to its treatment and prevention. Heliyon.

[8] Lima M., Teixeira-Santos R., Gomes L.C., Faria S.I., Valcarcel J., Vázquez J.A., Cerqueira M.A., Pastrana L., Bourbon A.I., Mergulhão F.J. (2021). Development of Chitosan-Based Surfaces to Prevent Single- and Dual-Species Biofilms of Staphylococcus aureus and Pseudomonas aeruginosa. Molecules.

[9] Mandakhalikar K.D., Chua R.R., Tambyah P.A. (2016). New Technologies for Prevention of Catheter Associated Urinary Tract Infection. Curr. Treat. Options Infect. Dis..

[10] Tunney M.M., Gorman S.P., Patrick S. (2002). Infection associated with medical devices. Int. J. Gen. Syst..

[11] Vertes A., Hitchins V., Phillips K.S. (2012). Analytical challenges of microbial biofilms on medical devices. Anal. Chem..

[12] Azevedo A.S., Almeida C., Melo L.F., Azevedo N.F. (2017). Impact of polymicrobial biofilms in catheter-associated urinary tract infections. Crit. Rev. Microbiol..

[13] Donlan R.M. (2001). Biofilms and device-associated infections. Emerg. Infect. Dis..

[14] Seif Eldein S.S., El-Temawy A.-E.-K.A., Ahmed E.H. (2013). Biofilm Formation by *E. coli* Causing Catheter Associated Urinary Tract Infection ( CAUTI ) in Assiut University Hospital. Egypt. J. Med. Microbiol..

[15] Niveditha S., Pramodhini S., Umadevi S., Kumar S., Stephen S. (2012). The isolation and the biofilm formation of uropathogens in the patients with catheter associated urinary tract infections (UTIs). J. Clin. Diagn. Res..

[16] Chen Q., Zhu Z., Wang J., Lopez A.I., Li S., Kumar A., Yu F., Chen H., Cai C., Zhang L. (2017). Probiotic *E. coli* Nissle 1917 biofilms on silicone substrates for bacterial interference against pathogen colonization. Acta Biomater..

[17] Vlamakis H., Kolter R. (2010). Biofilms. Cold Spring Harb. Perspect. Biol..

[18] Donlan R.M. (2002). Biofilms: Microbial life on surfaces. Emerg. Infect. Dis..

[19] Flemming H.C., Wingender J. (2010). The biofilm matrix. Nat. Rev. Microbiol..

[20] Rabin N., Zheng Y., Opoku-Temeng C., Du Y., Bonsu E., Sintim H.O. (2015). Biofilm formation mechanisms and targets for developing antibiofilm agents. Future Med. Chem..

[21] Schembri M.A., Klemm P. (2001). Biofilm formation in a hydrodynamic environment by novel FimH variants and ramifications for virulence. Infect. Immun..

[22] Lawrence E.L., Turner I.G. (2005). Materials for urinary catheters: A review of their history and development in the UK. Med. Eng. Phys..

[23] Stærk K., Grønnemose R.B., Palarasah Y., Kolmos H.J., Lund L., Alm M., Thomsen P., Andersen T.E. (2021). A Novel Device-Integrated Drug Delivery System for Local Inhibition of Urinary Tract Infection. Front. Microbiol..

[24] Chen M., Yu Q., Sun H. (2013). Novel strategies for the prevention and treatment of biofilm related infections. Int. J. Mol. Sci..

[25] Zhu Z., Wang Z., Li S., Yuan X. (2019). Antimicrobial strategies for urinary catheters. J. Biomed. Mater. Res.—Part A.

[26] Singha P., Locklin J., Handa H. (2017). A review of the recent advances in antimicrobial coatings for urinary catheters. Acta Biomater..

[27] AGN FAO, Nutrition and Consumer Protection Div, WHO, Geneva (2006). FAO Probiotics in Food: Health and Nutritional Properties and Guidelines for Evaluation.

[28] Fioramonti J., Theodorou V., Bueno L. (2003). Probiotics: What are they? What are their effects on gut physiology?. Best Pract. Res. Clin. Gastroenterol..

[29] Gogineni V.K., Morrow L.E. (2013). Probiotics: Mechanisms of action and clinical applications. J. Probiotics Heal..

[30] Aoudia N., Rieu A., Briandet R., Deschamps J., Chluba J., Jego G., Garrido C., Guzzo J. (2016). Biofilms of *Lactobacillus plantarum* and *Lactobacillus fermentum*: Effect on stress responses, antagonistic effects on pathogen growth and immunomodulatory properties. Food Microbiol..

[31] Muñoz M., Mosquera A., Alméciga-Díaz C.J., Melendez A.P., Sánchez O.F. (2012). Fructooligosaccharides metabolism and effect on bacteriocin production in *Lactobacillus* strains isolated from ensiled corn and molasses. Anaerobe.

[32] de Melo Pereira G.V., de Oliveira Coelho B., Magalhães Júnior A.I., Thomaz-Soccol V., Soccol C.R. (2018). How to select a probiotic? A review and update of methods and criteria. Biotechnol. Adv..

[33] Carr F.J., Chill D., Maida N. (2002). The lactic acid bacteria: A literature survey. Crit. Rev. Microbiol..

[34] Prabhurajeshwar C., Chandrakanth R.K. (2017). Probiotic potential of lactobacilli with antagonistic activity against pathogenic strains: An in vitro validation for the production of inhibitory substances. Biomed. J..

[35] Bermudez-Brito M., Plaza-Díaz J., Muñoz-Quezada S., Gómez-Llorente C., Gil A. (2012). Probiotic mechanisms of action. Ann. Nutr. Metab..

[36] Ng S.C., Hart A.L., Kamm M.A., Stagg A.J., Knight S.C. (2009). Mechanisms of action of probiotics: Recent advances. Inflamm. Bowel Dis..

[37] Khalighi A., Behdani R., Kouhestani S. (2016). Probiotics: A comprehensive review of their classification, mode of action and role in human nutrition. Probiotics Prebiotics Hum. Nutr. Health..

[38] Ray Mohapatra A., Jeevaratnam K. (2019). Inhibiting bacterial colonization on catheters: Antibacterial and antibiofilm activities of bacteriocins from *Lactobacillus plantarum* SJ33. J. Glob. Antimicrob. Resist..

[39] Hasslöf P., Hedberg M., Twetman S., Stecksén-Blicks C. (2010). Growth inhibition of oral *mutans* streptococci and *candida* by commercial probiotic lactobacilli—An *in vitro* study. BMC Oral Health.

[40] Vahedi Shahandashti R., Kasra Kermanshahi R., Ghadam P. (2016). The inhibitory effect of bacteriocin produced by *Lactobacillus acidophilus* ATCC 4356 and *Lactobacillus plantarum* ATCC 8014 on planktonic cells and biofilms of *Serratia marcescens*. Turkish J. Med. Sci..

[41] Jalilsood T., Baradaran A., Song A.A.L., Foo H.L., Mustafa S., Saad W.Z., Yusoff K., Rahim R.A. (2015). Inhibition of pathogenic and spoilage bacteria by a novel biofilm-forming *Lactobacillus* isolate: A potential host for the expression of heterologous proteins. Microb. Cell Fact..

[42] Carvalho F.M., Teixeira-Santos R., Mergulhão F.J.M., Gomes L.C. (2021). Effect of *Lactobacillus plantarum* Biofilms on the Adhesion of *Escherichia coli* to Urinary Tract Devices. Antibiotics.

[43] Sambanthamoorthy K., Feng X., Patel R., Patel S., Paranavitana C. (2014). Antimicrobial and antibiofilm potential of biosurfactants isolated from lactobacilli against multi-drug-resistant pathogens. BMC Microbiol..

[44] Kaur S., Sharma P., Kalia N., Singh J., Kaur S. (2018). Anti-biofilm properties of the fecal probiotic lactobacilli against *Vibrio* spp.. Front. Cell. Infect. Microbiol..

[45] Otero M.C., Nader-Macías M.E. (2006). Inhibition of *Staphylococcus aureus* by H2O2-producing *Lactobacillus gasseri* isolated from the vaginal tract of cattle. Anim. Reprod. Sci..

[46] Barzegari A., Kheyrolahzadeh K., Mahdi S., Khatibi H., Sharifi S., Memar M.Y., Vahed S.Z. (2020). The battle of probiotics and their derivatives against biofilms. Infect. Drug Resist..

[47] Carvalho F.M., Teixeira-Santos R., Mergulhão F.J.M., Gomes L.C. (2021). The use of probiotics to fight biofilms in medical devices: A systematic review and meta-analysis. Microorganisms.

[48] Carvalho F.M., Teixeira-Santos R., Mergulhão F.J.M., Gomes L.C. (2021). Targeting biofilms in medical devices using probiotic cells: A systematic review. AIMS Mater. Sci..

[49] Jeong D., Kim D.H., Song K.Y., Seo K.H. (2018). Antimicrobial and anti-biofilm activities of *Lactobacillus kefiranofaciens* DD2 against oral pathogens. J. Oral Microbiol..

[50] Cadieux P., Watterson J.D., Denstedt J., Harbottle R.R., Puskas J., Howard J., Gan B.S., Reid G. (2003). Potential application of polyisobutylene-polystyrene and a *Lactobacillus* protein to reduce the risk of device-associated urinary tract infections. Colloids Surf B Biointerfaces.

[51] Reid G., Tieszer C. (1994). Use of lactobacilli to reduce the adhesion of *Staphylococcus aureus* to catheters. Int. Biodeterior. Biodegrad..

[52] Gomes L.C., Silva L.N., Simões M., Melo L.F., Mergulhão F.J. (2015). *Escherichia coli* adhesion, biofilm development and antibiotic susceptibility on biomedical materials. J. Biomed. Mater. Res. Part A.

[53] Azevedo A.S., Almeida C., Gomes L.C., Ferreira C., Mergulhão F.J., Melo L.F., Azevedo N.F. (2017). An *in vitro* model of catheter-associated urinary tract infections to investigate the role of uncommon bacteria on the *Escherichia coli* microbial consortium. Biochem. Eng. J..

[54] Tan Y., Leonhard M., Moser D., Ma S., Schneider-Stickler B. (2018). Inhibitory effect of probiotic lactobacilli supernatants on single and mixed non-albicans *Candida* species biofilm. Arch. Oral Biol..

[55] Matsubara V.H., Wang Y., Bandara H.M.H.N., Mayer M.P.A., Samaranayake L.P. (2016). Probiotic lactobacilli inhibit early stages of *Candida albicans* biofilm development by reducing their growth, cell adhesion, and filamentation. Appl. Microbiol. Biotechnol..

[56] Rossoni R.D., de Barros P.P., de Alvarenga J.A., de Camargo Ribeiro F., dos Santos Velloso M., Fuchs B.B., Mylonakis E., Jorge A.O.C., Junqueira J.C. (2018). Antifungal activity of clinical *Lactobacillus* strains against *Candida albicans* biofilms: Identification of potential probiotic candidates to prevent oral candidiasis. Biofouling.

[57] Fernández Ramírez M.D., Smid E.J., Abee T., Nierop Groot M.N. (2015). Characterisation of biofilms formed by *Lactobacillus plantarum* WCFS1 and food spoilage isolates. Int. J. Food Microbiol..

[58] Jaffar N., Ishikawa Y., Mizuno K., Okinaga T., Maeda T. (2016). Mature biofilm degradation by potential probiotics: *Aggregatibacter actinomycetemcomitans* versus *Lactobacillus* spp.. PLoS ONE.

[59] Song Y.G., Lee S.H. (2017). Inhibitory effects of *Lactobacillus rhamnosus* and *Lactobacillus casei* on *Candida* biofilm of denture surface. Arch. Oral Biol..

[60] Fayol-Messaoudi D., Berger C.N., Coconnier-Polter M.H., Liévin-Le Moal V., Servin A.L. (2005). pH-, lactic acid-, and non-lactic acid-dependent activities of probiotic lactobacilli against *Salmonella enterica* serovar *typhimurium*. Appl. Environ. Microbiol..

[61] Lin X., Chen X., Tu Y., Wang S., Chen H. (2017). Effect of probiotic lactobacilli on the growth of *Streptococcus mutans* and multispecies biofilms isolated from children with active caries. Med. Sci. Monit..

[62] Maldonado-Barragán A., Caballero-Guerrero B., Lucena-Padrós H., Ruiz-Barba J.L. (2013). Induction of bacteriocin production by coculture is widespread among plantaricin-producing *Lactobacillus plantarum* strains with different regulatory operons. Food Microbiol..

[63] Klaenhammer T.R. (1988). Bacteriocins of lactic acid bacteria. Biochimie.

[64] McMillan A., Dell M., Zellar M.P., Cribby S., Martz S., Hong E., Fu J., Abbas A., Dang T., Miller W. (2011). Disruption of urogenital biofilms by lactobacilli. Colloids Surf. B Biointerfaces.

[65] Cadieux P.A., Burton J.P., Devillard E., Reid G. (2009). *Lactobacillus* by-products inhibit the growth and virulence of uropathogenic *Escherichia coli*. J. Physiol. Pharmacol..

[66] Morais I.M.C., Cordeiro A.L., Teixeira G.S., Domingues V.S., Nardi R.M.D., Monteiro A.S., Alves R.J., Siqueira E.P., Santos V.L. (2017). Biological and physicochemical properties of biosurfactants produced by *Lactobacillus jensenii* P6A and *Lactobacillus gasseri* P65. Microb. Cell Fact..

[67] Ceresa C., Tessarolo F., Caola I., Nollo G., Cavallo M., Rinaldi M., Fracchia L. (2015). Inhibition of *Candida albicans* adhesion on medical-grade silicone by a *Lactobacillus*-derived biosurfactant. J. Appl. Microbiol..

[68] Sharma D., Saharan B.S. (2016). Functional characterization of biomedical potential of biosurfactant produced by *Lactobacillus helveticus*. Biotechnol. Reports.

[69] Song H., Zhang J., Qu J., Liu J., Yin P., Zhang G., Shang D. (2019). *Lactobacillus rhamnosus* GG microcapsules inhibit *Escherichia coli* biofilm formation in coculture. Biotechnol. Lett..

[70] Ahn K.B., Baik J.E., Park O.J., Yun C.H., Han S.H. (2018). *Lactobacillus plantarum* lipoteichoic acid inhibits biofilm formation of *Streptococcus mutans*. PLoS ONE.

[71] Kim A.R., Ahn K.B., Yun C.H., Park O.J., Perinpanayagam H., Yoo Y.J., Kum K.Y., Han S.H. (2019). *Lactobacillus plantarum* lipoteichoic acid inhibits oral multispecies biofilm. J. Endod..

[72] Teanpaisan R., Piwat S., Dahlén G. (2011). Inhibitory effect of oral *Lactobacillus* against oral pathogens. Lett. Appl. Microbiol..

[73] Alexandre Y., Le Berre R., Barbier G., Le Blay G. (2014). Screening of *Lactobacillus* spp. for the prevention of *Pseudomonas aeruginosa* pulmonary infections. BMC Microbiol..

[74] Todorov S., Gotcheva B., Dousset X., Onno B., Ivanova I. (2000). Influence of growth medium on bacteriocin production in *Lactobacillus plantarum* ST31. Biotechnol. Biotechnol. Equip..

[75] Brooks T., Keevil C.W. (1997). A simple artificial urine for the growth of urinary pathogens. Lett. Appl. Microbiol..

[76] Rodrigues L., Banat I.M., Teixeira J., Oliveira R. (2006). Biosurfactants: Potential applications in medicine. J. Antimicrob. Chemother..

[77] Fracchia L., Cavallo M., Giovanna M., Banat I.M., Ghista D.N. (2012). Biosurfactants and Bioemulsifiers Biomedical and Related Applications—Present Status and Future Potentials. Biomedical Science, Engineering and Technology.

[78] Cerqueira L., Oliveira J.A., Nicolau A., Azevedo N.F., Vieira M.J. (2013). Biofilm formation with mixed cultures of *Pseudomonas aeruginosa/Escherichia coli* on silicone using artificial urine to mimic urinary catheters. Biofouling.

[79] Leroy F., De Vuyst L. (2001). Growth of the Bacteriocin-Producing *Lactobacillus sakei* Strain CTC 494 in MRS Broth is Strongly Reduced Due to Nutrient Exhaustion: A Nutrient Depletion Model for the Growth of Lactic Acid Bacteria. Appl. Environ. Microbiol..

[80] Gomes M., Gomes L.C., Teixeira-Santos R., Pereira M.F.R., Soares O.S.G.P., Mergulhão F.J. (2021). Optimizing CNT Loading in Antimicrobial Composites for Urinary Tract Application. Appl. Sci..

