# Supplementary material for: Using Lactobacilli to Fight Escherichia coli and Staphylococcus aureus Biofilms on Urinary Tract Devices
Source: Antibiotics (Basel). 2021 Dec 14;10(12):1525. doi: 10.3390/antibiotics10121525 (PMC8698619; doi:10.3390/antibiotics10121525)
Supplement: Supplementary file 1 [file antibiotics-10-01525-s001.zip › antibiotics-1425089-supplementary.pdf]

antibiotics

MDPI

Supplementary Material

1. Detection of Antimicrobial Compounds by Growth Inhibition on Agar Plate

1.1. Disk diffusion method

The antimicrobial activity of viable and lysed *Lactobacillus plantarum* and *Lactobacillus rhamnosus* cell suspensions and cell-free supernatants (CFSs) against *Staphylococcus aureus* was assessed by the disk diffusion method. The viable cell suspensions of both probiotics were directly used from the overnight cultures, whereas the lysed cell suspensions were obtained by sonication of 10 mL of overnight cultures with at least 3 short cycles of 15 sec (Sonopuls HD 2200 probe; Bandelin Electronic, Germany) in ice, followed by intervals of 30 sec for cooling, in order to release into the extracellular medium all the antimicrobial substances not secreted by the cells. Probiotic CFSs were obtained by centrifugation (3202 × g, 10 min) of each overnight sample (viable and lysed), followed by filtration through a 0.2-µm pore-size syringe filter (Whatman, Germany). Then, 10 µL of each sample was dropped in 6-mm-diameter disks placed on the top of Luria-Bertani agar plates previously swabbed with *S. aureus* (10^8 CFU·ml^{-1}) and plates were incubated for 24 h at 37 °C. Sterile De Man, Rogosa and Sharpe broth was used as the negative control. After incubation, the diameter of the inhibition zone around the disks was measured. The experiments included two independent experiments performed in triplicate.

1.2. Inhibition zones of S. aureus

Table S1. The inhibitory activity of viable and lysed *L. plantarum* and *L. rhamnosus* suspensions and cell-free supernatants on the growth of *S. aureus*.

**Sample**	**Diameter of inhibition zone (mm)**	
	L. plantarum	***L. rhamnosus***
Control	6.0 ± 0.0	
Cell suspension (viable)	9.7 ± 0.8	9.8 ± 0.8
Cell-free supernatant (viable)	6.8 ± 0.5	8.3 ± 0.8
Cell suspension (lysed)	9.6 ± 0.8	8.8 ± 0.4
Cell-free supernatant (lysed)	7.8 ± 1.3	7.3 ± 0.5

Inhibition zone values, inclusive of disk diameter of 6 mm, present the mean and standard deviation of two independent experiments performed in triplicate.
